# Non-Fullerene Organic Semiconductor ITIC as a Redox Mediator in Electrochemical Glucose Biosensors

**DOI:** 10.3390/s25247535

**Published:** 2025-12-11

**Authors:** Maurício A. P. Papi, Victor G. Scheidweiler, Sandra de Melo Cassemiro, Leni C. Akcelrud, Marcio F. Bergamini, Luiz Humberto Marcolino-Junior

**Affiliations:** 1 Laboratório de Sensores Eletroquímicos (LabSensE), Departamento de Química, Universidade Federal do Paraná (UFPR), Curitiba 81531-980, PR, Brazil; 2 Paulo Scarpa Polymer Laboratory (LaPPS), Departamento de Química, Universidade Federal do Paraná (UFPR), Curitiba 81531-980, PR, Brazil

**Keywords:** glucose analysis, non-fullerene mediator, electrochemical biosensing, screen-printed electrodes, portable devices

## Abstract

ITIC’s superior electron-accepting capacity and efficient oxygen reduction motivated the design of a sensor to enhance sensitivity, selectivity, and stability over conventional oxygen-dependent or fullerene-based systems. As oxygen acts as the terminal reagent in enzymatic glucose oxidation, we developed an ITIC-mediated glucose oxidase (GOx) biosensor on glassy carbon (GCE) and screen-printed carbon electrodes (SPCE). ITIC, a non-fullerene organic semiconductor, was drop-cast onto the electrode to catalyze oxygen reduction, followed by GOx immobilization in a chitosan matrix. Scanning electron microscopy (SEM) confirmed uniform, ultrathin coatings without significant morphological changes upon ITIC and GOx deposition. Electrochemical studies (cyclic (CV) and differential pulse voltammetry (DPV)) revealed a distinct ITIC reduction peak at –0.7 V (vs. Ag/AgCl) and a glucose-dependent current decrease, consistent with mediated electron transfer during enzymatic oxidation. Under optimized conditions, the GCE-based biosensor showed a sensitivity of 10.7 μA L mmol^−1^, a linear dynamic range (LDR) of 0.10–1.00 mmol L^−1^, and detection (LOD)/quantification (LOQ) limits of 0.02 and 0.06 mmol L^−1^, respectively. The SPCE device displayed sensitivity (3.8 μA L mmol^−1^) and maintained excellent linearity (R^2^ > 0.99) with LOD and LOQ of 0.05 and 0.16 mmol L^−1^. Both platforms showed good precision (RSD < 5%) and reliable recovery in deproteinized plasma and artificial tears (90–104%). The superior performance of the GCE is attributed to higher ITIC loading, faster electron transfer, and reduced background current, while the SPCE offers a low-cost, disposable format with sufficient analytical performance for point-of-care glucose monitoring.

## 1. Introduction

Diabetes mellitus is one of the most prevalent chronic diseases worldwide and a major cause of morbidity and mortality [1]. Accurate and continuous monitoring of blood glucose levels is therefore essential for effective disease management and prevention of severe complications. Electrochemical biosensors remain among the most promising technologies for glucose detection due to their simplicity, sensitivity, and suitability for portable and wearable devices [2]. Glucose oxidase (GOx) catalyzes glucose oxidation while reducing oxygen, the basis of first-generation glucose sensors. However, these devices suffer from oxygen dependence, high overpotentials, and interference by electroactive species [3]. Second-generation sensors introduced mediators such as ferrocene [4], osmium complexes [5], and fullerenes [6], but challenges with stability, cost, and solubility still remain.

Recent advances in organic electronics have introduced non-fullerene acceptors (NFAs) as versatile alternatives to fullerene derivatives in optoelectronic systems. Among them, ITIC (2,2′-[[6,6,12,12-Tetrakis(4-hexylphenyl)-6,12-dihydrodithieno [2,3-d:2′,3′-d′]-s-indaceno [1,2-b:5,6-b′]dithiophene-2,8-diyl]bis[methylidyne(3-oxo-1H-indene-2,1(3H)-diylidene)]]bis[propanedinitrile]), Figure S1, has emerged as one of the most successful NFAs, originally developed for organic photovoltaics. ITIC’s molecular architecture, centered on a fused-ring electron-deficient core, imparts key characteristics for its application: the extended π-conjugation promotes efficient electron mobility, the high electron affinity favors reversible reduction and effective electron acceptance, and the inherent chemical robustness ensures long-term operational stability [7,8]. Compared to fullerene-based acceptors, ITIC and its derivatives (e.g., ITIC-Th, ITIC-4F) show improved molecular packing, charge mobility, and tunable optoelectronic properties, making them attractive for electrochemical applications [9].

A particularly relevant feature of ITIC is its catalytic activity toward the oxygen reduction reaction (ORR). The effective electron mediation of ITIC arises from its optimal energy-level alignment and regenerative redox cycle. In this process, the reduced form of ITIC, a stable radical anion (ITIC^−^), generated at the electrode surface, acts as an efficient electron shuttle between the reduced oxygen and the electrode. Signal amplification occurs as ITIC^−^ is subsequently reoxidized by dissolved oxygen, and this O_2_-dependent regeneration of the mediator is crucial for sustaining the redox cycling and enhancing the current response [10]. This property positions ITIC as a promising redox mediator for enzymatic glucose biosensors, as it enhances the sensitivity of oxygen-based detection while minimizing interference from competing species. Furthermore, its robust fused-ring structure ensures stability under operational conditions, and its processability enables integration into flexible substrates, supporting the development of wearable continuous glucose monitoring devices [11].

In this work, we propose the development of an electrochemical glucose sensor based on ITIC combined with glucose oxidase. By exploiting ITIC’s superior electron-accepting capacity and its ability to mediate oxygen electroreduction, the proposed sensor aims to achieve improved sensitivity, selectivity, and operational stability compared to traditional oxygen-dependent or fullerene-mediated systems. This approach demonstrates the potential of non-fullerene organic semiconductors to advance biosensing technologies and opens new pathways for next-generation glucose monitoring platforms [12].

## 2. Materials and Methods

### 2.1. Chemicals and Materials

3,9-bis(2-methylene-(3-(1,1-dicyanomethylene)-indanone))-5,5,11,11-tetrakis (4-hexylphenyl)-dithieno [2,3-d:2′,3′-d′]-s-indaceno [1,2-b:5,6-b′] dithiophene (ITIC) was purchased from Aldrich (São Paulo, Brazil). Polyvinyl chloride (PVC) sheets served as electrode substrates, while vinyl adhesive sheets (0.12 mm thickness) were used to produce stencils. Neutral cure silicone was applied as an insulating material. Ethanol and neutral soap were used for cleaning, and ultrapure water (resistivity ≥ 18.2 MΩ·cm) was used in all aqueous preparations.

### 2.2. Reagents and Solutions

A 1.0 μmol L^−1^ ITIC solution in toluene was prepared. Glucose oxidase (GOx, from *Aspergillus niger*) was dissolved at 10 mg mL^−1^ in acetate buffer (pH 5.5). Chitosan (0.1% *m*/*v*) was prepared by dissolving in 1% (*m*/*v*) acetic acid, followed by dilution with ultrapure water.

### 2.3. Fabrication of Screen-Printed Carbon Electrodes (SPCEs) and Electrode Modification

Electrodes were fabricated via a stencil-based method [13]. Electrode patterns were designed in Silhouette Studio^®^ 4.4, cut using a Silhouette Cameo 3 plotter (São Paulo, Brazil), and transferred onto PVC substrates. Substrates were cleaned with neutral soap, rinsed with ultrapure water, dried under nitrogen, and degreased with ethanol. Conductive ink (SunChemical—C2130925D1) was spread through the stencil using a squeegee, forming uniform electrode films. After stencil removal, electrodes were cured overnight at 60 °C. The working electrode area (ϕ = 3 mm) was then insulated with neutral cure silicone to ensure reproducibility. Figure S2 illustrates the main steps involved in the construction of the screen-printed electrodes (SPEs).

**Figure 1:**
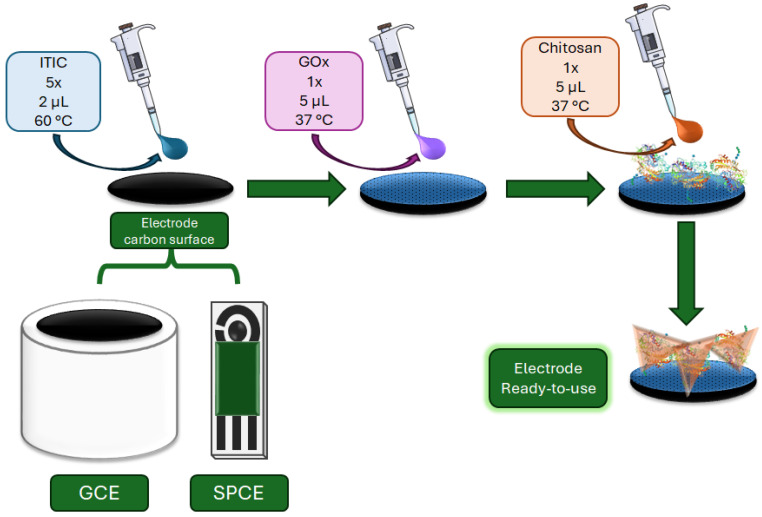
Schematic procedure of electrodes (GCE or SPCE) modification with ITIC, GOx, and chitosan, showing sequential drop-casting and biolayer stabilization.

Figure 1 illustrates the schematic procedure for electrode modification. Glassy carbon electrodes (GCE, ϕ = 3 mm) were polished with 0.3 and 0.05 μm alumina, sonicated in water and ethanol, and dried under nitrogen. Modification was carried out by sequential drop-casting: ITIC solution (2 μL, 1.0 μmol L^−1^) was deposited and dried at 60 °C, repeated five times. GOx solution (5 μL, 10 mg mL^−1^) was added and dried at 37 °C. Chitosan solution (5 μL, 0.1% *m*/*v*) was applied as a final coating and dried at 37 °C. The same procedure was followed for SPCE modification. For both platforms used (GCE and SPCE), the ITIC loading was 8.40 ng·cm^−2^.

### 2.4. Electrochemical Measurements

Electrochemical experiments were performed on a μAutolab Type III potentiostat/galvanostat (Metrohm Autolab, Utrecht, The Netherlands) with NOVA 2.1.7 software. A three-electrode setup was used: modified GCE (working electrode), Ag/AgCl (3 mol L^−1^ KCl, reference electrode), and Pt wire (auxiliary electrode); for SPCE, all three electrodes were composed of carbon ink. Measurements were conducted in 0.1 mol L^−1^ acetate buffer (pH 5.5).

Differential pulse voltammetry (DPV) was used for glucose quantification under optimized parameters: initial potential 0.9 V, final potential −1.2 V, step −5 mV, amplitude 0.15 V, modulation time 0.015 s, interval 0.05 s, scan rate 0.1 V s^−1^. These conditions were used for calibration and sample analysis.

To improve signal stability, conditioning was tested by applying potentials (0.3–1.1 V vs. Ag/AgCl) for 60–120 s before measurements. Calibration curves were constructed in triplicate using 0.1–1.0 mmol L^−1^ glucose solutions in acetate buffer.

### 2.5. Biological Sample Analysis

To evaluate the practical applicability and selectivity of the biosensor, tests were performed using two biological matrices: human blood commercial plasma and artificial tear fluid.

Blood plasma: Commercial Human AB plasma samples were clarified by centrifugation at 2900× *g* (7200 rpm) for 10 min at 4 °C using a fixed-angle rotor (*r* ≈ 5 cm). After centrifugation, proteins were precipitated by the addition of trifluoroacetic acid (TFA) to a final concentration of 0.1% (*v*/*v*), followed by incubation for 10 min on ice. The precipitate was pelleted by centrifugation at 2900× *g* (7200 rpm) for 10 min at 4 °C and the supernatant was carefully collected. The supernatant was neutralized to pH 5.5 by the gradual addition of acetate buffer (1.0 M, pH 6.0) while monitoring pH with a calibrated pH meter. After neutralization, samples were diluted 1:40 in 0.1 mol·L^−1^ PBS (pH 7.4) prior to analysis. The TFA deproteinization step proved effective, yielding clear supernatants with no visible turbidity, ensuring minimal protein interference in subsequent analyses. Prior to analysis, samples were inspected visually for turbidity. If turbidity was observed, the procedure was repeated, and the samples were subsequently centrifuged at 2900× *g* (7200 rpm) for 5 min.

Artificial tears: A synthetic tear solution was prepared (albumin 5 mg, urea 6 mg, NH_4_Cl 6.6 mg, MgCl_2_ 0.25 mg, KCl 7.4 mg, NaCl 58.4 mg in 10 mL ultrapure water) following [14]. For analysis, glucose (0.3–0.6 mmol L^−1^) was spiked into 1.66 mL aliquots, diluted to 5 mL with buffer before measurement.

Selectivity was assessed against typical interferents. In plasma, ascorbic acid, dopamine, and uric acid were tested at 250 µmol L^−1^, which can be considered representative of physiological levels [14]. In artificial tears, each component was individually examined for interference with glucose detection [15].

## 3. Results and Discussion

### 3.1. Morphological and Electrochemical Characterization of the Modified Electrodes

**Figure 2:**
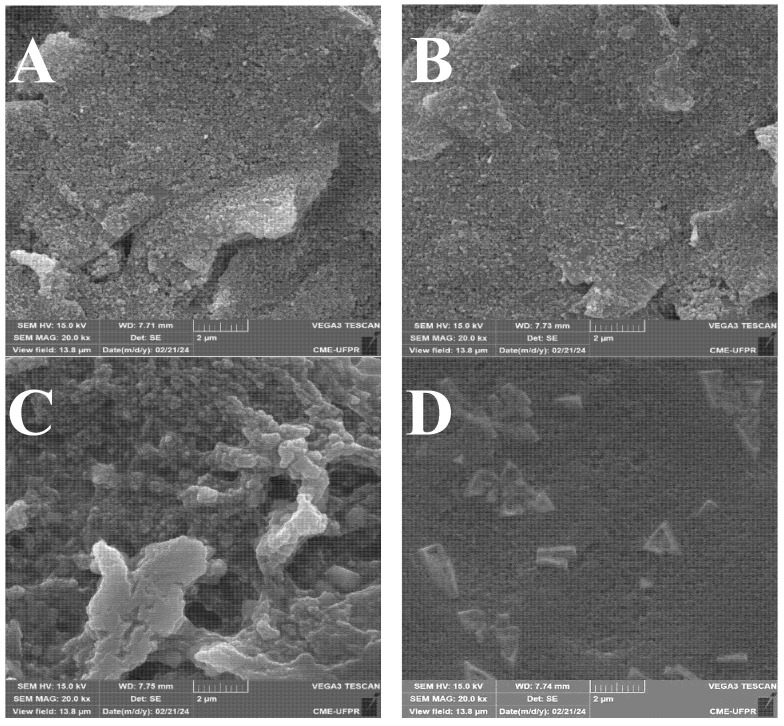
Representative SEM images of the SPCE: (**A**) bare, (**B**) after ITIC modification, (**C**) after glucose oxidase (GOx) immobilization, and (**D**) after chitosan coating.

The surface changes in the electrode during the modification steps were first evaluated by SEM. Representative SEM images of bare electrodes displayed a smooth surface with polishing striations and minor pits (Figure 2A). After ITIC deposition, no significant topographical changes were verified, suggesting the formation of a thin conformal layer below SEM resolution (Figure 2B). Immobilization of glucose oxidase (GOx) introduced globular aggregates consistent with protein deposition (Figure 2C), while the subsequent addition of a chitosan film produced a more heterogeneous surface, with arrow-like structures and particulates ranging from 200 to 500 nm (Figure 2D). These features indicate that chitosan not only stabilizes the biolayer but also increases surface roughness, favoring enzyme retention [15].

**Figure 3:**
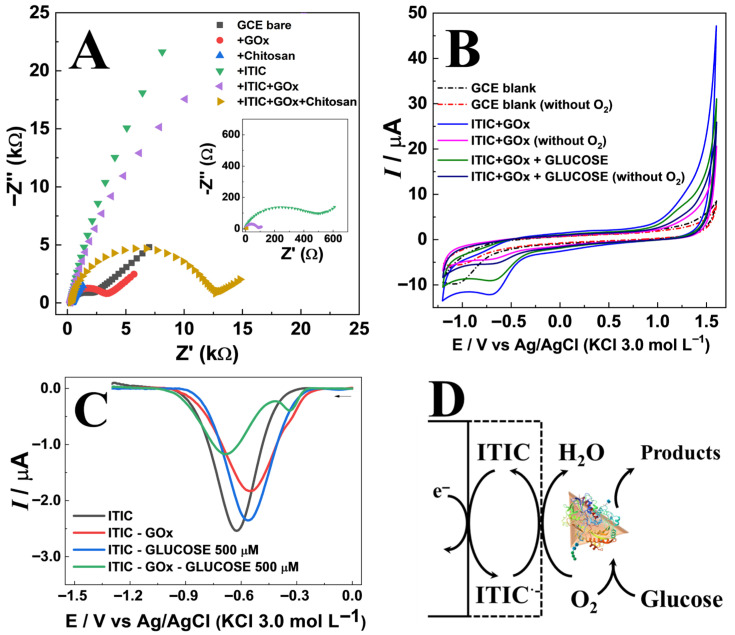
(**A**) Electrochemical impedance spectroscopy (EIS) spectra obtained for the GCE at each modification stage. (**B**) Cyclic voltammograms (CV) of unmodified and ITIC-modified electrodes in aerated and deaerated acetate buffer (pH 5.5), in the absence and presence of 1.0 mmol L^−1^ glucose. (**C**) Differential Pulse voltammetry obtained for different sensors arranged in the presence and absence of 500 μmol L^−1^ of glucose. (**D**) Schematic representation of the ITIC/GOx/chitosan electrode architecture, illustrating mediator–enzyme interaction and oxygen reduction pathway.

Electrochemical characterization (Figure 3) of the sensor construction was performed using electrochemical impedance spectroscopy (EIS), cyclic voltammetry (CV), and differential pulse voltammetry (DPV). The EIS spectra were fitted using a modified Randles circuit, and the values obtained after each assembly step are reported in Table S1. Results confirmed the impact of each modification step on charge transfer resistance (Rct) as shown in Figure 3A. The GCE bare electrode exhibited a moderate semicircle in Nyquist plots (Rct = 1.81 kΩ), which increased slightly after GOx immobilization (Rct ~ 2.95 kΩ) due to the insulating nature of the protein [16]. Chitosan forms a porous thin film that slightly increases the Rct (8.29 kΩ). In contrast, ITIC coating produced a pronounced increase in Rct (35.7 kΩ), reflecting the semiconducting character of the organic layer [17]. The intermediate Rct value (16.2 kΩ) observed for the final ITIC–GOx–chitosan assembly indicates that the synergistic contribution of these components creates a surface environment that favors more efficient enzyme–mediator interactions [18]. In addition, the Rs values correspond to the high-frequency intercept of the Nyquist plot and represent not only the true solution resistance but also contributions from cell geometry, electrode–contact resistance, and wiring. Thus, the slight changes observed after surface modification (e.g., with chitosan) arise mainly from interfacial/wetting and contact effects rather than from variations in the bulk electrolyte conductivity.

Cyclic voltammetry (CV) of the ITIC-modified electrode in acetate buffer (pH 5.5) showed a characteristic cathodic peak at −0.7 V vs. Ag/AgCl (Figure 3B), assigned to ITIC reduction [8]. In aerated solutions, ITIC significantly enhanced cathodic currents over a wide potential range, confirming its electrocatalytic activity toward oxygen reduction [19]. Upon deaeration, the current decreased markedly, reinforcing the role of ITIC as an oxygen reduction mediator. The influence of pH on the voltammetric response was investigated in the range of 4.0–10.0 (Section S1). A linear shift in the cathodic peak potential with pH (≈−17 mV/pH) indicated the participation of protons in the redox process (Figure S3A). The highest and most stable current responses were observed at pH 5.0, which is close to the optimal value for glucose oxidase (GOx) activity (Figure S3B) [19].

Differential pulse voltammetry (DPV) further (Figure 3C) revealed that glucose addition in the presence of GOx led to a consistent decrease in ITIC peak current, attributed to enzymatic oxygen consumption [19]. The proposed response mechanism follows an EC′ (electrochemical–chemical catalytic) pathway. Initially, ITIC undergoes electrochemical reduction (E step), as described by ITIC + e^−^ → ITIC^•−^. The reduced form, ITIC^•−^, subsequently catalyzes the chemical reduction of oxygen (C′ step) according to 4ITIC^•−^ + O_2_ + 4H^+^ → 4ITIC + 2H_2_O, thereby regenerating the original ITIC species [20,21]. This regeneration enables continuous cycling, producing a current proportional to the oxygen concentration. In the presence of glucose, oxygen is consumed by the enzymatic reaction catalyzed by GOx [22,23,24] leading to a decrease in O_2_ concentration and, consequently, a lower voltammetric signal corresponding to the ITIC reduction current at approximately −0.7 V (Figure 3D).

Taking into consideration all results from morphological and electrochemical characterization, the successful construction of the ITIC-based electrode is confirmed. The semiconductor acts both as a mediator for oxygen reduction and as a transducer of oxygen depletion during enzymatic glucose oxidation, corroborating its suitability for biosensing applications [17,25,26]. These features provide the foundation for the biosensor’s analytical application, discussed in the next section.

### 3.2. Analytical Performance of the ITIC-Based Biosensor

**Figure 4:**
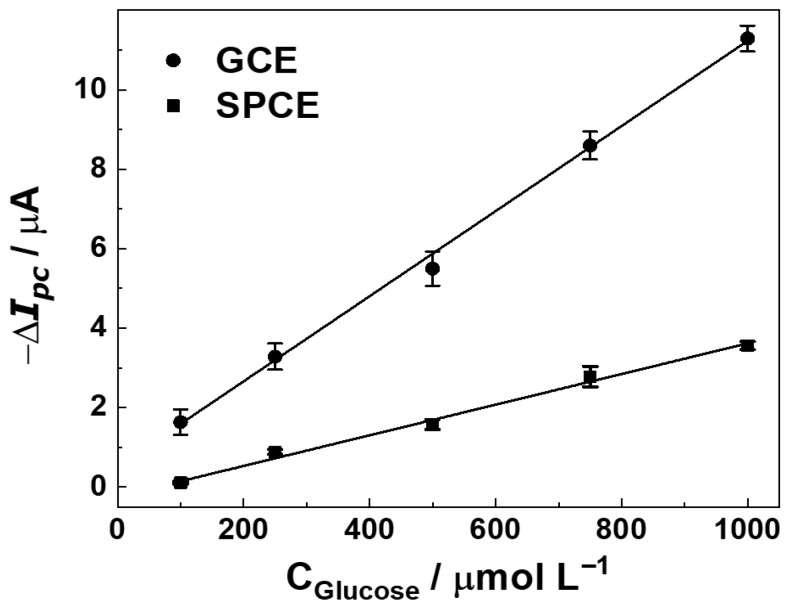
Calibration curves of ITIC/GOx biosensors fabricated on GCE and SPCE platforms, showing the current response to increasing glucose concentrations (100–1000 µmol L^−1^) in acetate buffer (pH 5.5).

The effects of conditioning potential and time (0.3–1.1 V) applied prior to each DPV scan were evaluated, as detailed in Section S2. Figure S4A, a potential of +0.9 V yielded the highest analytical signal (ΔI, current change upon glucose addition) and improved reproducibility. The duration of the conditioning step was also optimized, with Figure S4B showing that 90 s provided the most stable and substantial current response. Electrode pretreatment promotes the stabilization of the modification layers, resulting in decreased current variability, reduced blank-signal fluctuations, and enhanced analytical sensitivity. Therefore, conditioning at +0.9 V for 90 s was adopted in all subsequent experiments to ensure a stable, pretreated electrode surface and to improve measurement precision and accuracy. Additionally, a systematic investigation of the electrochemical sensor construction was carried out prior to evaluating its analytical performance for glucose determination. Key experimental parameters, such as the amount of ITIC modifier (Figure S5A), were optimized to ensure maximum sensitivity and reliable operation of the biosensor. Under the optimized DPV conditions, the ITIC/GOx-modified electrodes (GCE and SPCE) exhibited a clear linear dependence of the cathodic current on glucose concentration (Figure 4).

Figure 4 illustrates the calibration curve obtained with the GCE, showing a linear range between 0.1 and 1.0 mmol L^−1^, described by the regression equation ΔI (µA) = 10.7 (±0.25) [Glucose] (mmol L^−1^) + 0.5 (±0.15), with R^2^ = 0.9984. Figure 4 presents the corresponding curve for SPCE, which also displayed linearity within the same range. Such high correlation coefficients confirm the robustness of ITIC as a redox mediator for quantitative glucose determination [3]. The LOD and LOQ, calculated as 3.3 × SD/slope and 10 × SD/slope, respectively [27], where SD is the standard deviation of blank measurement (*n* = 3) and slope corresponds to the angular coefficient of the analytical curve. Differences in sensitivity can be attributed to the surface characteristics of the electrodes. The polished glassy carbon electrode (GCE), after successive drop-casting cycles, forms a relatively thick and uniform ITIC film that increases the density of redox-active sites per geometric area due to its higher electrical conductivity. In contrast, the porous carbon-ink matrix and heterogeneous microstructure of the SPCE result in a thinner and less homogeneous mediator layer, thereby decreasing the slope of its calibration curve. There was observed LOD and LOQ of 0.02 and 0.06 mmol L^−1^ for GCE, while for SPCE the values were 0.05 and 0.17 mmol L^−1^, respectively. These sensitivity levels are relevant for physiological monitoring. In blood plasma, where glucose typically ranges from 4.0 to 5.4 mmol L^−1^, a simple 40-fold dilution places concentrations into the 0.1–0.135 mmol L^−1^ window, within the sensor’s linear range. In tear fluid, where glucose levels vary from 0.1 to 0.6 mmol L^−1^, minimal dilution is sufficient for accurate detection [28]. The obtained LODs are comparable to those of state-of-the-art glucose sensors based on carbon nanotubes or MOFs, which report detection limits between 0.01 and 0.1 mmol L^−1^ but often require complex fabrication [29].

To contextualize the analytical performance of the proposed ITIC-based biosensor, Table 1 summarizes representative enzymatic and non-enzymatic electrochemical approaches reported for glucose determination, highlighting their linear ranges, detection limits, and sample applications. Non-enzymatic sensors such as Multiplex/GLU/SPE, RCE-Cu^2+^, and SPE/CNT-Ni show good sensitivity for direct glucose oxidation, with LODs down to the micromolar range, but often face challenges with selectivity and surface fouling in biofluids. Enzymatic systems like PB/AB-CPME, RGO–C60/GOx/GCE, and NF/MWCNTs-HFs-GOx/GCE employ different mediators to improve selectivity and extend linearity, achieving LODs between 1 and 10 μmol·L^−1^ in biological samples. By comparison, the ITIC/GOx electrodes developed here achieved linear detection from 0.1 to 1.0 mmol·L^−1^ with LODs of 20 μmol·L^−1^ (GCE) and 50 μmol·L^−1^ (SPE), positioning ITIC as a promising mediator that balances sensitivity, selectivity, and the practicality of disposable platforms. In addition, the LOD values obtained for the ITIC-based biosensors (20 and 50 µmol L^−1^ for GCE and SPCE, respectively) fall within the range reported for other enzymatic systems and are comparable to, or only slightly higher than, those of the best-performing fullerene- or nanocarbon-based mediators listed in Table 1.

Although both electrodes followed the same ITIC/GOx modification protocol, their analytical performance differed. The GCE provided a sensitivity of 10.7 μA mmol^−1^ L, nearly three times higher than the 3.8 μA mmol^−1^ L observed for the SPCE. Several factors account for this difference: (1) successive drop-casting cycles on GCE produced a thicker and more homogeneous ITIC film, maximizing the density of electroactive sites; (2) the atomically smooth and conductive GCE surface facilitated faster electron transfer and reduced interfacial resistance, while the heterogeneous porous microstructure of SPCE increased impedance and dispersed the mediator unevenly; (3) the larger electrolyte volume in GCE measurements favored semi-infinite diffusion, enhancing analyte availability, whereas the small volume in SPCE imposed diffusion constraints; and (4) the planar GCE showed lower capacitive background currents, improving signal-to-noise, while the rough SPCE surface exhibited elevated double-layer capacitance, reducing slope and sensitivity [36,37,38]. These structural and electrochemical differences explain the improved performance of GCE, although the SPCE retains important advantages in disposability and low fabrication cost [39].

To evaluate reproducibility (Section S3, Figure S6), six independently prepared ITIC/GOx-GCE electrodes were fabricated on different days and tested with 0.50 mmol L^−1^ glucose under optimized conditions. The relative standard deviation (RSD) of 8.22% reflects good inter-electrode reproducibility, consistent with values reported for nanomaterial-modified SPEs [38]. The inclusion of chitosan as a biocompatible matrix contributed to the structural stability of the film, as has been demonstrated in biosensors for physiological fluids [40].

Overall, the ITIC-based biosensors demonstrated linear response in the 0.1–1.0 mmol L^−1^ range, with detection limits down to 0.02 mmol L^−1^. These parameters confirm the suitability of the approach for monitoring glucose in diluted blood and tear fluid, while offering competitive performance compared to inorganic nanomaterial-based sensors [29]. The results reinforce ITIC’s potential as a low-cost organic semiconductor mediator that combines electrocatalytic activity toward oxygen with compatibility for enzyme immobilization.

### 3.3. Selectivity and Application in Biological Samples

The selectivity of electrochemical biosensors is a critical factor for their application in complex matrices [41]. Therefore, the potential interference from common electroactive species was first investigated. In the case of blood, ascorbic acid (AA), dopamine (DA), and uric acid (UA) are typical interferents due to their overlapping redox activity with many mediators [42]. When tested in the presence of 0.25 mmol L^−1^ glucose, these species caused minimal deviations in the current signal at their physiological concentrations, confirming that they do not significantly compromise glucose detection. For artificial tear fluid, inorganic salts such as KCl, MgCl_2_, NH_4_Cl, NaCl, and urea represent potential interferents [43]. Their individual addition to glucose-containing solutions caused negligible changes in the DPV response, further demonstrating the good selectivity of the ITIC/GOx-based biosensor (Section S4, Figure S7A,B).

After an investigation related to selectivity, the biosensor was applied to the determination of glucose in biological samples. In human plasma, direct addition produced strong matrix effects, likely due to protein fouling or other electroactive constituents. To address this, plasma was pretreated with trifluoroacetic acid (TFA) for protein precipitation [41]. After pretreatment, the biosensor determined a glucose concentration of 4.73 (±0.39) mmol L^−1^ in a sample containing 4.99 mmol L^−1^, corresponding to a recovery of 94.8% and a relative error of 5.2% (*n* = 3).

In artificial tears, glucose was spiked at 0.3 and 0.6 mmol L^−1^ (*n* =3). The biosensor quantified 0.27 (±0.03) mmol L^−1^ (90.0% recovery) and 0.62 (±0.05) mmol L^−1^ (103.3% recovery), respectively [42]. Comparable tests on SPCE-based sensors yielded recovery values of 104.1% and 107.4% for spiked tears, and 97.7% for plasma, confirming good accuracy across both electrode platforms. Thus, the ITIC/GOx biosensor exhibited strong selectivity against common interferents and reliable performance in complex matrices such as plasma and tears. The biosensor exhibited a stable electrochemical response for up to one week when stored in buffer solution at 10 °C (*n* = 3), indicating satisfactory short-term stability. These findings highlight the robustness of ITIC as a redox mediator in glucose biosensing, validating its potential for bioanalytical applications [11,43].

## 4. Conclusions

This work demonstrates the successful use of ITIC, an organic semiconductor traditionally explored in photovoltaics, as a redox mediator for electrochemical biosensing. By integrating ITIC into both glassy carbon electrodes and screen-printed carbon electrodes, the study shows that this material not only supports efficient electron transfer but also provides stability and selectivity in complex biological fluids. Importantly, the adaptation of ITIC to SPCEs highlights its potential for low-cost, disposable, and portable sensing platforms, which are highly desirable for point-of-care diagnostics. Beyond glucose detection, the outcomes of this work establish ITIC as a versatile component in bioelectrochemical devices, bridging advances in organic electronics and analytical chemistry. Its compatibility with scalable printing technologies further strengthens its relevance for developing sustainable and user-friendly diagnostic tools. The advances reported here set the stage for extending ITIC-based strategies to other clinically and environmentally relevant targets, paving the way for innovative disposable sensors with broad applicability.

## Figures and Tables

**Table 1:** Analytical performance of enzymatic and non-enzymatic electrochemical sensors, including the proposed ITIC-based biosensor, reported for glucose determination.

Sensor	Technique	LDR	LOD	Matrix Tested	Ref.
Non-Enzymatic					
Multiplex/GLU/SPE ^a^	Amperometry	0.10–2.50 mmol L^−1^	8.50 μmol L^−1^	Mineral water, coconut water, and commercial juice	[13]
RCE-Cu ^2+ b^	Amperometry	0.005–1.0 mmol L^−1^	1.8 μmol L^−1^	Human blood plasma	[30]
SPE/CNT-Ni ^c^	Amperometry	0.025–1 μmol L^−1^	3.9 μmol L^−1^	Human blood plasma	[31]
NiAB-CPME ^d^	Amperometry	0.005–0.1 µmol L^−1^	0.137 µmol L^−1^	Human saliva, Human blood serum	[32]
PPy-AgNPs/GCE ^e^	Amperometry	0.025–2.5 μmol L^− 1^	3.6 μmol L^−1^	Human saliva	[33]
**Enzymatic**					
PB/AB-CPME ^f^	Amperometry	0.05–5.0 mmol L^−1^	0.94 μmol L^−1^	Human saliva, Human blood serum	[24]
PTB-GOx/G ^g^	Amperometry	0.075–7.5 mmol L^−1^	22.2 µmol L^−1^	Human tear	[14]
RGO–C60/GOx/GCE ^h^	Amperometry	0.1–12.5 mmol L^−1^	35 μmol L^−1^	Human blood serum	[34]
NF/MWCNTs-HFs-GOx/GCE ^i^	Square Wave Voltammetry	0.02–0.25 mmol L^−1^ and 0.25–4.0 mmol L^−1^	9.93 μmol L^−1^	Human Blood Serum	[6]
Chit/GOD-HF/GCE ^j^	Amperometry	0.05–1.0 mmol L^−1^ and 3.0–10.0 mmol L^−1^	5 μmol L^−1^	Human Blood Serum	[35]
ITIC/GOx/GCE	Differential Pulse Voltammetry	0.1–1.0 mmol L^−1^	20 μmol L^−1^	Human Blood Plasma; Artificial Tear Fluid	This work
ITIC/GOx/SPCE	Differential Pulse Voltammetry	0.1–1.0 mmol L^−1^	50 μmol L^−1^	Human Blood Plasma; Artificial Tear Fluid	This work

^a^ Multiplex/GLU/SPE Multiplex Glucose Screen-Printed Electrode; ^b^ RCE-Cu^2+^ Resin Composed Electrode with Copper ion; ^c^ SPE/CNT-Ni Screen-Printed Electrode modified with Carbon nanotube and Nickel; ^d^ NiAB-CPME nickel ions supported at activated biochar carbon paste electrode; ^e^ PPy-AgNPs/GCE polypyrrole silver nanocomposite in glassy carbon electrode; ^f^ PB/AB-CPME Prussian Blue Activated Biochar Carbon Paste Modified Electrode; ^g^ PTB-GOx Poly(toluidine Blue), Glucose Oxidase on Graphite Electrode; ^h^ RGO–C60/GOx/GCE Reduced Graphene Oxide/Fullerene/Glucose Oxidase on Glassy Carbon Electrode; ^i^ NF/MWCNTs-HFs-GOx/GCE Nafion, Multi-walled carbon nanotubes, Hydroxy Fullerene, Glucose Oxidase (GOx) modified glassy carbon electrode; ^j^ Chit/GOD-HF/GCE chitosan (Chit) membrane, glucose oxidase, hydroxyl fullerenes on Glassy Carbon Electrode; LDR—Linear Dynamic Range; LOD—Limit of Detection.

## Data Availability

The data that support the findings of this study are available from the corresponding author upon reasonable request, due to privacy and legal restrictions.

## Supplementary Materials

The following supporting information can be downloaded at: https://www.mdpi.com/article/10.3390/s25247535/s1, Figure S1: ITIC (C94H82N4O2S4) molecular structure; Figure S2: Fabrication of lab-made screen-printed carbon electrodes (SPCEs), illustrating stencil design, conductive ink deposition, and final insulating layer; Table S1: Compound parameter values extracted from the modified Randles’ equivalent circuit used for EIS fitting; Section S1: Effect of pH; Figure S3: Influence of buffer pH on the electrochemical response of ITIC-modified electrodes: (A) linear shift of the anodic peak potential with pH, and (B) variation of anodic peak current as a function of pH; Section S2. Development of Electrochemical Method; Figure S4: Optimization of electrode preconditioning. (A) Influence of applied potential (0.3–1.1 V) on analytical signal amplitude (ΔI). (B) Influence of preconditioning time (60–120 s) on signal stability, with optimum at +0.9 V for 90 s; Figure S5: Optimization of modifier loading on electrode performance. (A) Voltammograms of each amount of ITIC and (B) Effect of ITIC deposition volume on signal intensity and reproducibility; Section S3: Precision and reproducibility; Figure S6: Precision and reproducibility of the ITIC/GOx/GCE biosensor. Six independently prepared electrodes tested with 0.50 mmol L^−1^ glucose; Section S4: Interference Studies; Figure S7: Selectivity of the ITIC/GOx biosensor in complex matrices. (A) Effect of common plasma interferents (ascorbic acid, dopamine, uric acid) on glucose response. (B) Effect of artificial tear constituents (KCl, MgCl2, NH4Cl, NaCl, urea) on glucose response, showing negligible interference.
