# Supplementary material for: Non-Fullerene Organic Semiconductor ITIC as a Redox Mediator in Electrochemical Glucose Biosensors
Source: Sensors (Basel). 2025 Dec 11;25(24):7535. doi: 10.3390/s25247535 (PMC12737306; doi:10.3390/s25247535)
Supplement: Supplementary file 1 [file sensors-25-07535-s001.zip › sensors-3983346-supplementary.pdf]

Supplementary Material

Non-Fullerene Organic Semiconductor ITIC as a Redox Mediator in Electrochemical Glucose Biosensors

M.A.P. Papi[a]; V. G. Scheidweiler[a]; S.M. Cassemiro[b]; L.C. Akcelrud[b]; M.F. Bergamini[a]; L.H. Marcolino-Junior[a*]*

Tabel of content

Figure S1: *ITIC ($C_{94}H_{82}N_4O_2S_4$) molecular structure.* ***1***

Figure S2: *Fabrication of lab-made screen-printed carbon electrodes (SPCEs), illustrating stencil design, conductive ink deposition, and final insulating layer.* ***1***

Table S1:*Compound parameter values extracted from the modified Randles' equivalent circuit used for EIS fitting* ***2***

Section S1: Effect of pH ***3***

Figure S3: *Influence of buffer pH on the electrochemical response of ITIC-modified electrodes: (A) linear shift of the anodic peak potential with pH, and (B) variation of anodic peak current as a function of pH.* ***3***

Section S2. Development of Electrochemical Method ***4***

Figure S4: *Optimization of electrode preconditioning. (A) Influence of applied potential (0.3–1.1 V) on analytical signal amplitude (ΔI). (B) Influence of preconditioning time (60–120 s) on signal stability, with optimum at +0.9 V for 90 s.* ***4***

Figure S5*: Optimization of modifier loading on electrode performance. (A) Voltammograms of each amount of ITIC and (B) Effect of ITIC deposition volume on signal intensity and reproducibility.* ***5***

Section S3: Precision and reproducibility ***6***

Figure S6: *Precision and reproducibility of the ITIC/GOx/GCE biosensor. Six independently prepared electrodes tested with 0.50 mmol L^{-1} glucose* ***6***

Section S4: Interference Studies ***7***

Figure S7: *Selectivity of the ITIC/GOx biosensor in complex matrices. (A) Effect of common plasma interferents (ascorbic acid, dopamine, uric acid) on glucose response. (B) Effect of artificial tear constituents (KCl, $MgCl_2$, NH_4Cl, NaCl, urea) on glucose response, showing negligible interference.* ***7***

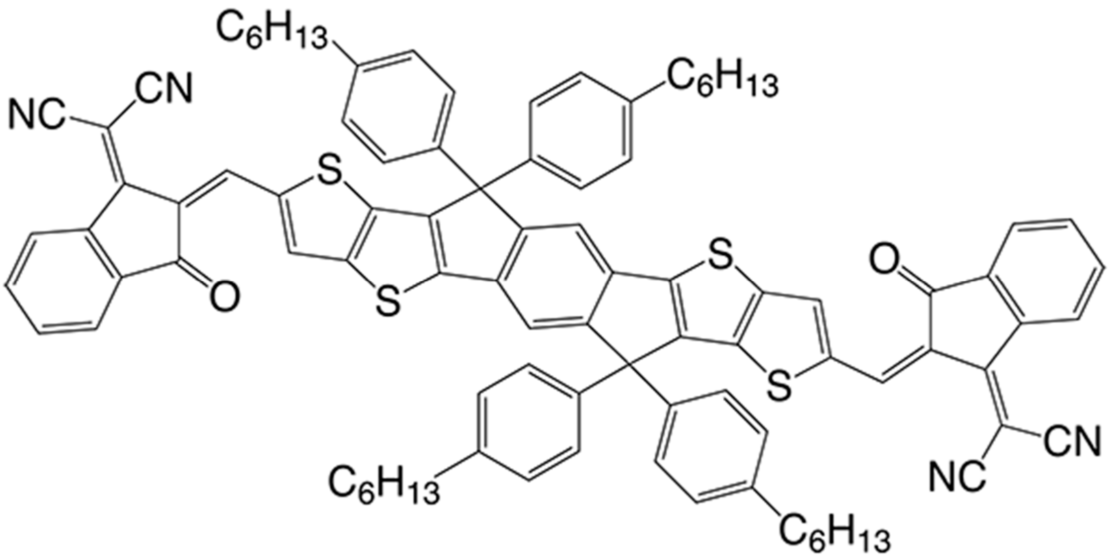

Figure S1: ITIC ($C_{94}H_{82}N_4O_2S_4$) molecular structure.

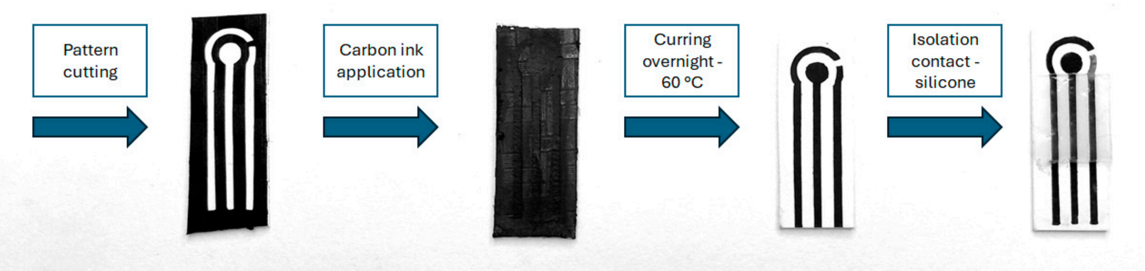

Figure S2: Fabrication of lab-made screen-printed carbon electrodes (SPCEs), illustrating stencil design, conductive ink deposition, and final insulating layer.

Table S1: Compound parameter values extracted from the modified Randles' equivalent circuit used for EIS fitting

Electrode	Rs / Ω	Rct / kΩ	CPE (Y_0 and N) / μS s^N	Walburg / μS $s^{1/2}$
GCE bare	241	1.81	3.840 / 0.869	182
+GOx	236	2.95	3.424 / 0.866	362
+Chitosan	337	8.28	746 / 0.641	44.2
+ITIC	241	35.7	1.178 / 0.756	127
+ITIC+GOx	259	10.5	0.765 / 0.881	318
+ITIC+GOx+Chitosan	244	16.2	0.752 / 0.865	382

Section S1: Effect of pH

The effect of solution pH on the ITIC-modified SPCE was evaluated through the shift of the cathodic peak potential (Ep). A linear dependence of −17 mV/pH indicated proton involvement in the electrode reaction, consistent with a complex multi-step mechanism. The cathodic peak current (Ip) was maximal at pH 5.0, which was therefore selected for further experiments, also aligning with the optimal activity range of glucose oxidase.

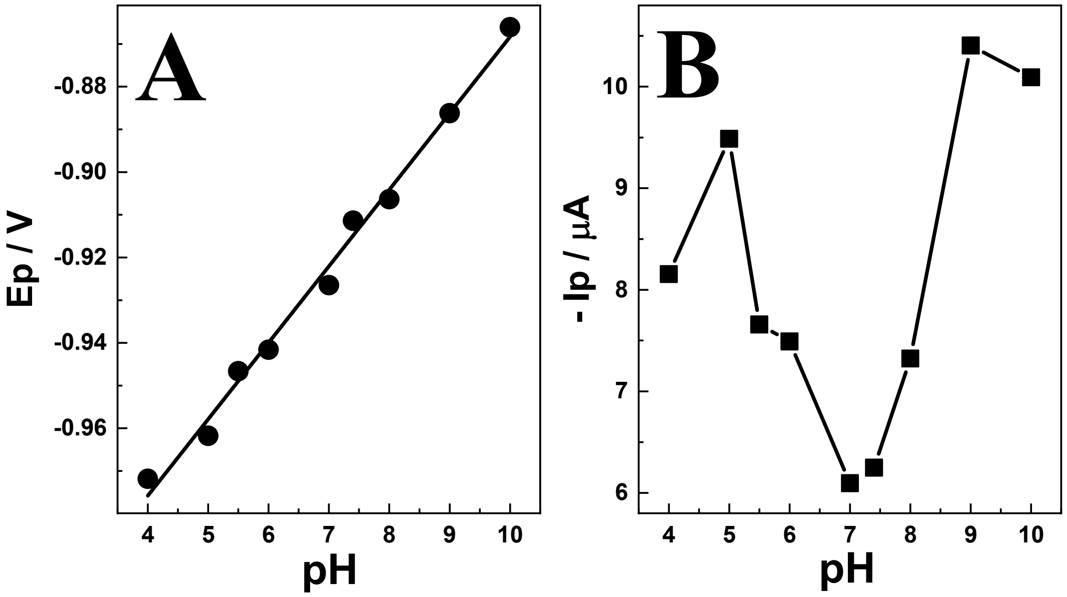

Figure S3: Influence of buffer pH on the electrochemical response of ITIC (electroactive mediator)-modified electrodes: (A) linear shift of the ITIC anodic peak potential with pH; (B) variation of ITIC anodic peak current as a function of pH.

Section S2. Development of Electrochemical Method

The conditioning potential applied prior to each DPV scan was varied between 0.3 and 1.1 V (Figure S4A). A potential of +0.9 V produced the highest analytical signal (ΔI) and improved reproducibility. The conditioning time was also optimized (Figure S4B), with 90 s yielding the most stable and consistent response. These conditions (+0.9 V for 90 s) were adopted for all subsequent experiments to ensure a stable electrode surface and reliable measurements.

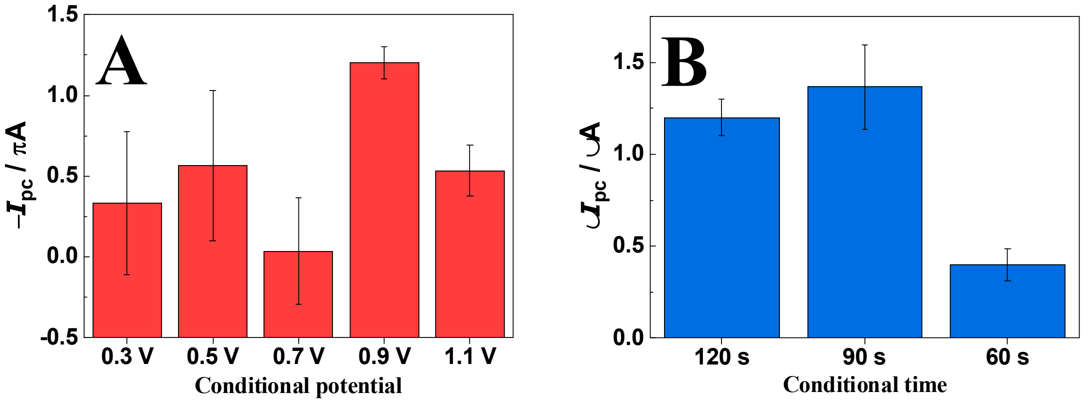

Figure S4: Optimization of electrode preconditioning for detection of the ITIC redox mediator signal. (A) Influence of applied potential (0.3–1.1 V) on the ITIC analytical signal amplitude (ΔI). (B) Influence of preconditioning time (60–120 s) on ITIC signal stability, with optimum at +0.9 V for 90 s.

The amount of ITIC deposited on the SPCE surface strongly affected sensor reproducibility. Two microliters of the ITIC solution yielded the best response, providing sufficient electroactive material without forming a resistive film (Figure S5A). The biosensor's sensitivity and linearity depended on the GOx concentration. A loading of 2.5 mg mL^{-1} produced a satisfactory linear response, whereas higher enzyme content (5.0 mg mL^{-1}) likely introduced diffusion resistance and signal saturation (Figure S5B).

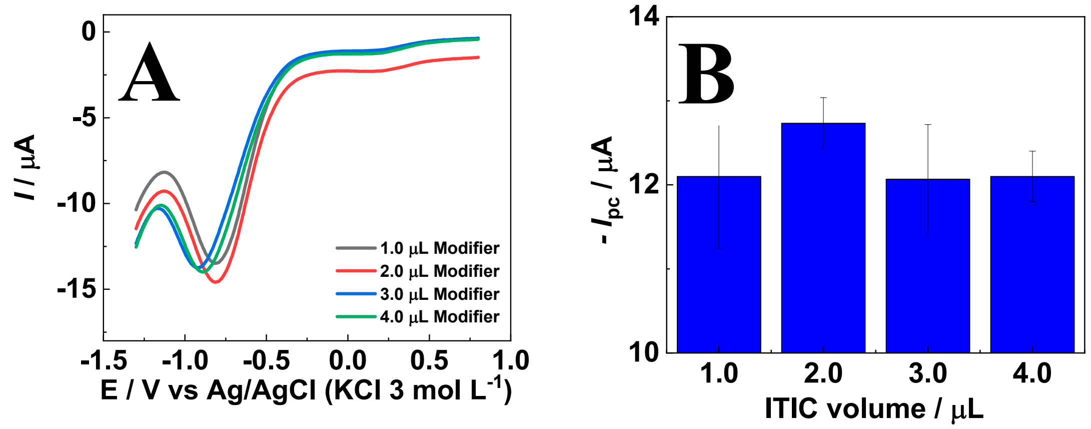

Figure S5: Optimization of modifier loading on the electrochemical performance of the ITIC electroactive mediator. (A) Voltammograms obtained for each deposited amount of ITIC. (B) Effect of ITIC deposition volume on signal intensity and reproducibility.

Section S3: Precision and reproducibility

The precision of the developed method was evaluated using six independently prepared ITIC/GOx-modified GCEs on different days, each tested with 0.50 mmol L^{-1} glucose under optimized conditions. Applying a conditioning potential of 0.9 V for 90 s stabilized the electrode surface, minimized signal drift, and improved reproducibility, as recommended for organic semiconductor-based electrodes where irregular molecular packing is common. The method achieved an RSD of 8.22%, comparable to SPEs modified with nanomaterials such as graphene oxide, underscoring ITIC's potential as a low-cost alternative to inorganic mediators.

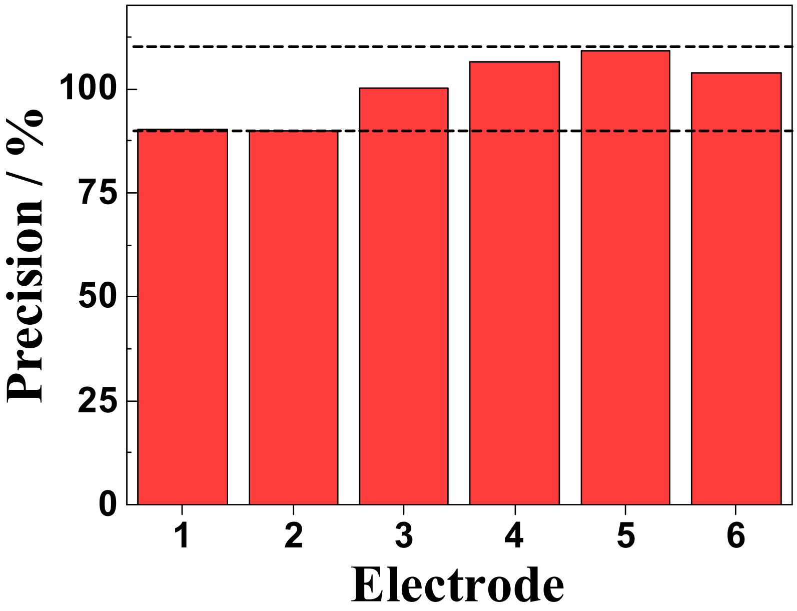

Figure S6: Precision and reproducibility of the ITIC/GOx/GCE biosensor. Six independently prepared electrodes tested with 0.50 mmol L^{-1} glucose.

Section S4: Interference Studies

Selectivity is a critical factor for electrochemical biosensors, especially in complex biological fluids. To evaluate potential interferences, common electroactive species from blood and tears were tested.

For blood, ascorbic acid (AA), dopamine (DA), and uric acid (UA) are typical interferents due to their overlapping redox potentials. Their addition to 0.25 mmol L^{-1} glucose solutions caused only minimal signal deviations (Figure S7A), confirming negligible interference at physiological levels. For tears, major components such as KCl, $MgCl_2$, NH_4Cl, NaCl, and urea were individually evaluated. As shown in Figure S7B, none produced significant changes in the glucose response, further demonstrating the sensor's selectivity.

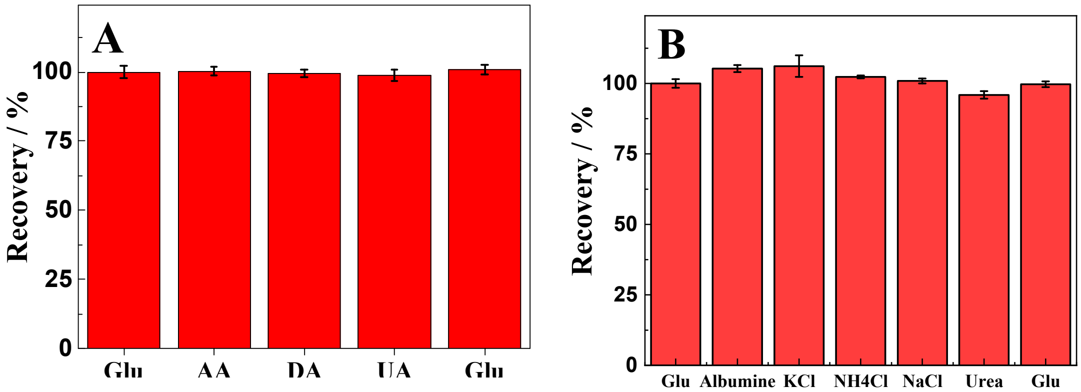

Figure S7: Selectivity of the ITIC/GOx biosensor in complex matrices. (A) Effect of common plasma interferents (ascorbic acid, dopamine, uric acid) on glucose response. (B) Effect of artificial tear constituents (KCl, MgCl2, NH_4Cl, NaCl, urea) on glucose response, showing negligible interference.

In summary, the biosensor incorporating ITIC as a redox mediator enabled accurate and reproducible glucose determination in treated human blood plasma and artificial tear fluid. Beyond validating ITIC's suitability for glucose biosensing, this study highlights its novel application as an organic semiconductor in bioanalytical chemistry.
